# Mutating the maternal haploid inducer gene *CsDMP* in cucumber produces haploids *in planta*

**DOI:** 10.1093/plphys/kiad600

**Published:** 2023-11-13

**Authors:** Shuai Yin, Sen Li, Lei Sun, Kexin Shi, Shanshan Fan, Xingwang Liu, Huazhong Ren

**Affiliations:** Department of Vegetable Science, College of Horticulture, China Agricultural University, Beijing 100193, China; Department of Vegetable Science, College of Horticulture, China Agricultural University, Beijing 100193, China; Department of Vegetable Science, College of Horticulture, China Agricultural University, Beijing 100193, China; Department of Vegetable Science, College of Horticulture, China Agricultural University, Beijing 100193, China; Department of Vegetable Science, College of Horticulture, China Agricultural University, Beijing 100193, China; Department of Vegetable Science, College of Horticulture, China Agricultural University, Beijing 100193, China; Sanya Institute of China Agricultural University, Sanya 572000, China; Department of Vegetable Science, College of Horticulture, China Agricultural University, Beijing 100193, China; Sanya Institute of China Agricultural University, Sanya 572000, China

## Abstract

Mutation of *DOMAIN OF UNKNOWN FUNCTION 679 MEMBRANE PROTEIN* in cucumber induces in vivo maternal haploids and suggests prospects for cucurbit breeding.

Dear Editor,

Doubled haploid (DH) technology could rapidly produce homozygous lines within 2 generations and is widely used in crop breeding (Jacquier et al. 2020). In vitro approaches, relying on androgenesis and gynogenesis, could produce haploids and are still popular in most plants but with lower efficiency. Nowadays, in vivo methods, such as haploid inducer, have been successfully applied to haploid induction. Haploid inducer avoids in vitro tissue culture steps and generates viable seeds with haploid embryos after simple crossing (Jacquier et al. 2020). Although haploid inducer lines have been widely utilized in maize (*Zea mays*) breeding (Wang et al. 2019), no haploid inducer has been identified, developed, or employed in cucumber (*Cucumis sativus* L., 2*n* = 2*x* = 14).

Cucumber, an important cucurbit crop widely cultivated worldwide, has rich nutrition and higher economic value. Breeding new cucumber varieties with high quality, resistance, and yield that meet the increasing market demand is essential. However, traditional breeding remains the predominant approach, and while the in vitro haploid induction method with lower efficiency has been used in cucumber breeding, there has been no development and utilization of an in vivo haploid inducer (Dong et al. 2016; Hooghvorst and Nogués 2020). Hence, developing haploid inducer is invaluable to the application of DH technology for expediting breeding processes in cucumber. Inactivation of several genes, such as *MATRILINEAL* (*MTL*)/*PHOSPHOLIPASE A1* (*PLA1*)/*NOT LIKE DAD* (*NLD*), *CENTROMERIC HISTONE3* (*CENH3*), and *DOMAIN OF UNKNOWN FUNCTION 679 MEMBRANE PROTEIN* (*DMP*), has been utilized to induce haploid production (Ravi and Chan 2010; Gilles et al. 2017; Kelliher et al. 2017; Liu et al. 2017; Zhong et al. 2019; Lv et al. 2020; Zhong et al. 2020; Wang et al. 2021). Nevertheless, *MTL*/*PLA1*/*NLD* is conserved in monocots but not dicots, and *CENH3* haploid induction engineering has only been successfully conduced in very limited crops. The *DMP* gene is highly conserved in both monocots and dicots, and mutation of *DMP* homologs could induce haploid and produce DHs in maize, tomato (*Solanum lycopersicon*), potato (*Solanum tuberosum* L.), watermelon (*Citrullus lanatus*), Arabidopsis (*Arabidopsis thaliana*), *Brassica napus*, *Nicotiana tabacum*, and *Medicago truncatula* (Zhong et al. 2019, 2020, 2022a, 2022b; Li et al. 2022; Wang et al. 2022; Zhang et al. 2022; Zhao et al. 2022; Chen et al. 2023; Tian et al. 2023). However, it is unknown whether the mutation of *DMP* homologs might be applied for haploid induction in cucumber.

Here, we identified 5 putative *DMP*-like genes in cucumber genome, 1 of which encodes CsDMP (CsaV3_1G028660), which shares the most similarity to ZmDMP with a protein sequence identity of 66.67% (Supplemental Table S1) and locates closest to watermelon, tomato, and Arabidopsis within the dicot evolutionary cluster (Supplemental Fig. S1). These findings suggested that *CsDMP* may have a conserved function. Similar to other homologs of DMP in crops, the fusion protein pSuper::CsDMP-EGFP colocalized with the marker CD-1007-mCherry on the plasma membrane in heterologous *Nicotiana benthamiana* leaves (Supplemental Fig. S2; Zhong et al. 2019; Wang et al. 2021). Next, we examined the expression pattern of *CsDMP* in cucumber. The reverse transcription quantitative PCR (RT-qPCR) results showed a higher expression level of *CsDMP* in male bud (at 10 d before flowering [DBF] stage) and mature pollen (at 0 DBF stage; Supplemental Fig. S3), hinting a potential function of *CsDMP* during gametophyte development in cucumber. To test this hypothesis, we mutated *CsDMP* via pKSE402 vector, a CRISPR-Cas9 toolkit that harbors a separated EGFP marker gene expression cassette triggered by a cauliflower mosaic virus (CaMV) 35S promoter (Fig. 1A). Two types of homozygous *csdmp* mutants with 1 bp insertion or 142 bp deletion that resulted in translational frame shifts and premature stop codons were generated in the local cultivar ‘xintaimici’ (wild type [WT]) cucumber background (Fig. 1B). Compared to WT, *csdmp* mutants showed lower pollen viability, a 10% reduced pollen germination rate, significantly reduced filled seed number, and increased aborted seed percentage (Fig. 1, C to E; Supplemental Fig. S4).

**Figure 1.**
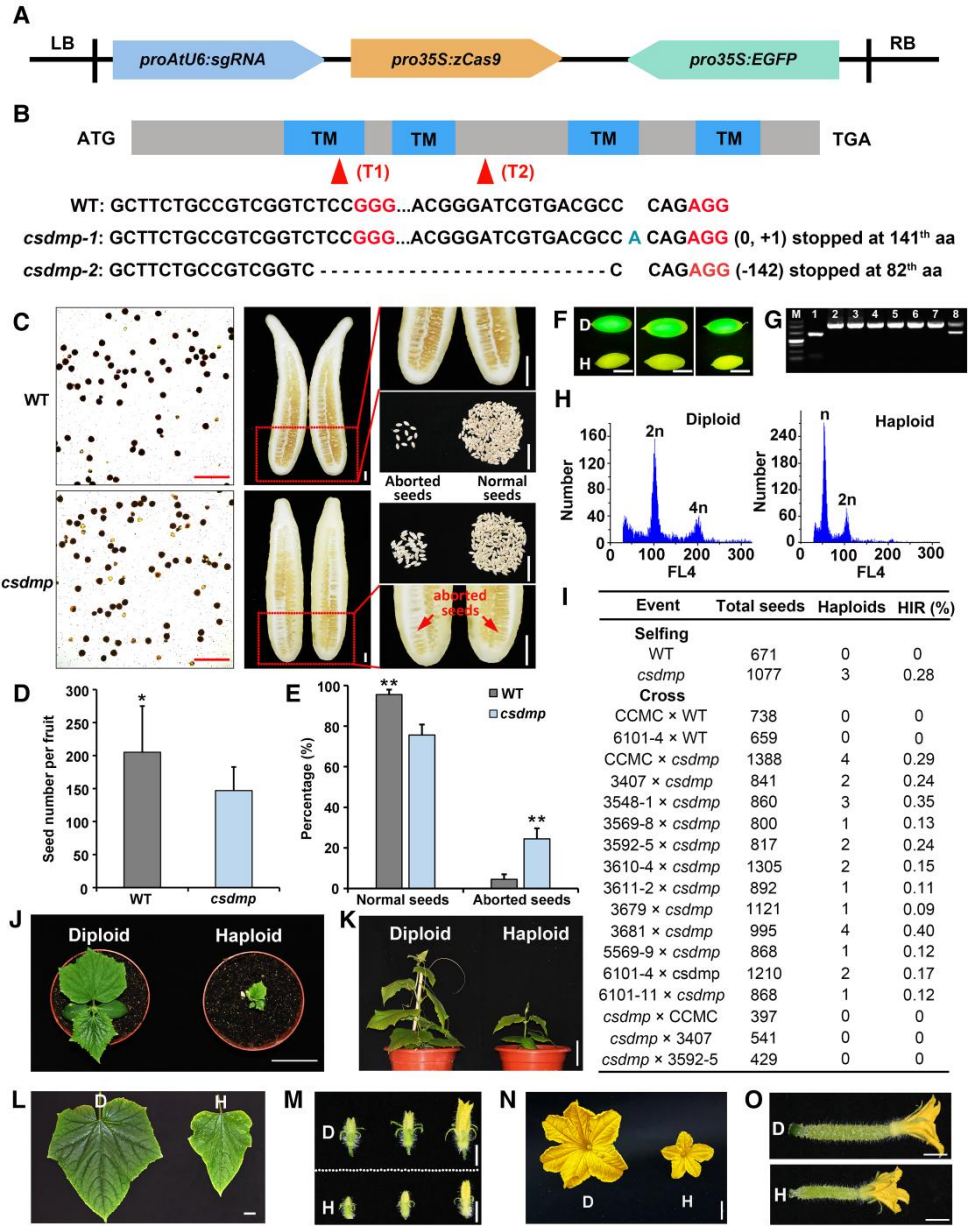
Mutation of *CsDMP* induces maternal haploid production in cucumber. **A)** The CRISPR-Cas9 editing vector targeting *CsDMP* gene. **B)** Schematic representation of *CsDMP* gene structure and the mutation types of *CsDMP*. Gray blocks: the coding region. Blue blocks: the 4 predicted transmembrane domains (TMs). Red triangles: the targeted regions. Mutation type: *csdmp-1*—1 bp (A) was inserted at 360 bp site from transcriptional start site (TSS); *csdmp-2*—142 bp (217 to 358 bp from TSS) was deleted. **C)** Mature pollen viability, seeds, and fruits from selfed WT ‘xintaimici’ and *csdmp* mutant. Red outline indicates the part of the fruit that has been enlarged. Red arrows indicate the aborted seeds. **D, E)** Quantification of seed number **D)** and seed phenotypes **E)** in fruits. The number of seeds was 2,786 for WT and 2,706 for *csdmp* mutant in **D)**. The number of normal seeds was 2,666 for WT and 2,057 for *csdmp* mutant, and the number of aborted seeds was 120 for WT and 649 for *csdmp* mutant in **E)**, respectively. The significant differences were analyzed by Student's *t* tests (*represents *P* ≤ 0.05 in **D**; **represents *P* ≤ 0.01 in **E**). These values were the means ± Sd. **F)** The seeds of diploid (D) and putative haploid (H) under fluorescent light. **G)** The putative haploid seedlings were genotyped with molecular marker. Left lane: DNA size marker; 1 to 8: PCR bands of *csdmp* mutant, cucumber materials CCMC, 3548-1, 3407, haploids from CCMC, 3548-1, and 3407, and hybrid diploid from CCMC × *csdmp*, respectively. **H)** Ploidy verification of diploid and haploid plants by flow cytometry. **I)** HIR of WT or *csdmp* mutant by selfing or outcrossing. **J)** Images of diploid and haploid seedlings. **K to O)** Phenotypic differences between diploid (D) and haploid (H) plants (whole plant **K**, leaf **L**, male buds **M**, male **N**, and female flower **O**) in cucumber. Scale bars: 5 cm (**C** [except pollen], **J**, **K**), 1 cm (**L to O**), 0.5 cm (**F**), and 100 *μ*m (**C** [pollen]).

To test whether the *csdmp* mutants can induce haploid production, we first selfed both WT and *csdmp* mutants. Three haploids, confirmed by flow cytometry (Fig. 1H), were obtained from a total of 1,077 progenies resulting from the selfing of *csdmp* mutants. However, no haploids were produced when selfing the WT (Fig. 1I). These results suggested that the mutation of *CsDMP* can trigger haploid embryo development in cucumber. To verify whether *csdmp* mutants induce maternal haploid production in cucumber, the *csdmp* T2 homozygous mutants, which are homozygous in both the mutant sites of *CsDMP* and the genome region of EGFP marker insertion, were used as male parent to cross with 12 cucumber materials from different genetic backgrounds, including 1 local cultivar (CCMC) and 11 inbred lines (listed in Fig. 1I and Supplemental Table S2). Given that inactivation of *DMP* induces maternal haploid might be due to the single fertilization that resulted from a defective sperm cell of *dmp* mutant (Jacquier et al. 2020), we thus screened the maternal haploids from the crosses’ progenies by using green fluorescence as an indicator. The offspring without green fluorescence were the putative haploids and then confirmed by molecular marker and flow cytometry (Fig. 1, F to H). In total, the average haploid induction rate (HIR) ranged from 0.09% to 0.40% (Fig. 1I; Supplemental Table S2). Furthermore, compared with diploid plants, the haploid plants are shorter and have smaller leaves, as well as male and female flowers (Fig. 1, J to O). We found no haploids derived from the 2 crosses of CCMC × WT and 6101-4 × WT. Also, no haploids were detected from the crosses’ progenies when *csdmp* mutant was used as a female (Fig. 1I). These results proved that *CsDMP* mutation efficiently induces in vivo maternal haploid production in genotype-independent manner in cucumber.

In conclusion, we have successfully demonstrated that *csdmp* mutants induce in vivo maternal haploids in the major cucurbit crop, cucumber. We also developed an effective and convenient method to identify haploid embryos by utilizing EGFP marker. This study represents the successful development of an in vivo haploid inducer in cucumber and provides insight into cucumber breeding. Further work is required to improve the efficiency of in vivo haploid inducer. Finally, we hope that extending this system will broaden the application of DH technology in cucurbit crop breeding.

## Supplementary Material

kiad600_Supplementary_Data

## Supplemental data

The following materials are available in the online version of this article.

**Supplemental Figure S1.** Phylogenetic analysis of CsDMP.

**Supplemental Figure S2.** Subcellular localization of CsDMP.

**Supplemental Figure S3.** The expression of *CsDMP* in different tissues.

**Supplemental Figure S4.** The pollen germination characteristics of *csdmp* mutant compared to WT.

**Supplemental Table S1.** *CsDMP*-like genes in cucumber.

**Supplemental Table S2.** The information of haploids from different crosses.

**Supplemental Table S3.** Primers used in this study.

**Supplemental Method S1.** Plant materials and growth conditions.

**Supplemental Method S2.** Vector construction and plant transformation.

**Supplemental Method S3.** Pollen viability determination and germination assay.

**Supplemental Method S4.** Haploid induction and identification.

**Supplemental Method S5.** Flow cytometry.

**Supplemental Method S6.** Phylogenetic analysis.

**Supplemental Method S7.** Subcellular localization.

**Supplemental Method S8.** RT-qPCR analysis.
