# Supplementary material for: Mutating the maternal haploid inducer gene CsDMP in cucumber produces haploids in planta
Source: Plant Physiol. 2023 Nov 13;194(3):1282–5. doi: 10.1093/plphys/kiad600 (PMC10904314; doi:10.1093/plphys/kiad600)
Supplement: kiad600_Supplementary_Data [file kiad600_supplementary_data.pdf]

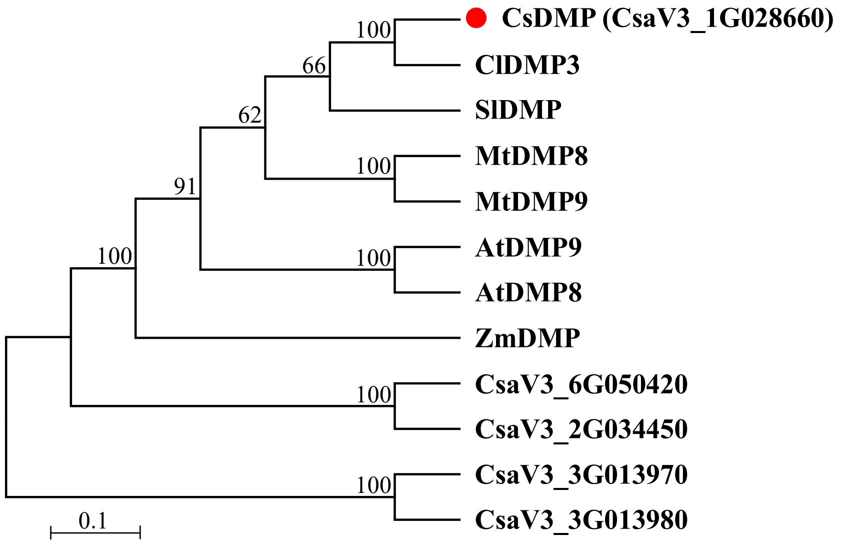

Supplemental Figure S1. Phylogenetic analysis of CsDMP. The DMP homologues from *Arabidopsis* (At), tomato (Sl), watermelon (Cl), *Medicago truncatula* (Mt), maize (Zm) and cucumber (Cs). The red circle hints the most similar *DMP* gene in cucumber with other DMP homologues.

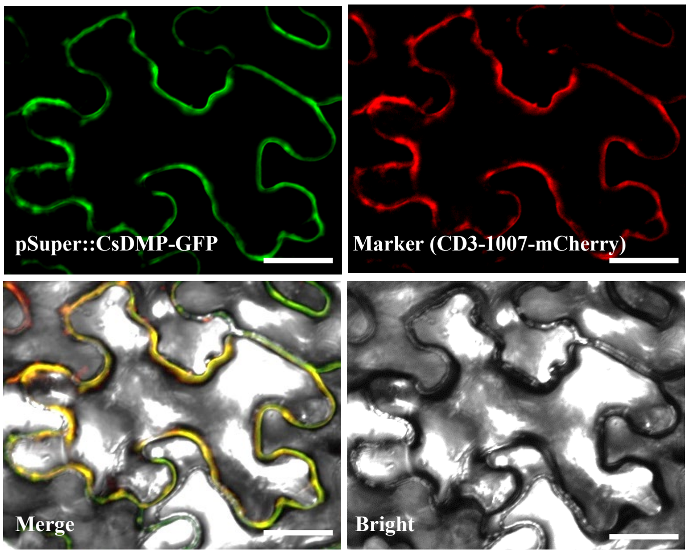

Supplemental Figure S2. Subcellular localization of CsDMP. The subcellular localization of CsDMP was performed in *Nicotiana benthamiana* leaves. The pm-rk CD3-1007-mCherry was used as a plasma membrane marker. Bar =50 μm.

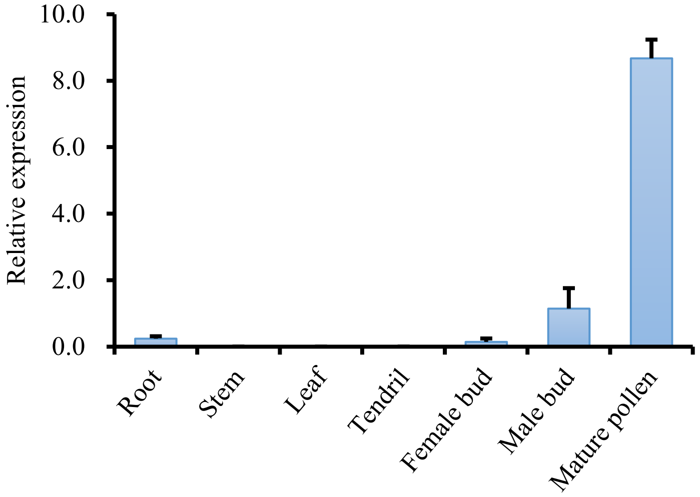

Supplemental Figure S3. The expression of *CsDMP* in different tissues. The expression levels of *CsDMP* in the tender root, stem, leaf, tendril, female and male buds at 10 days before flowering (DBF) stage, and mature pollen at 0DBF stage in cucumber. The values are the means ± s.d.. Three biological (each sample involved three technical repetitions) replicates were carried out for expression analysis.

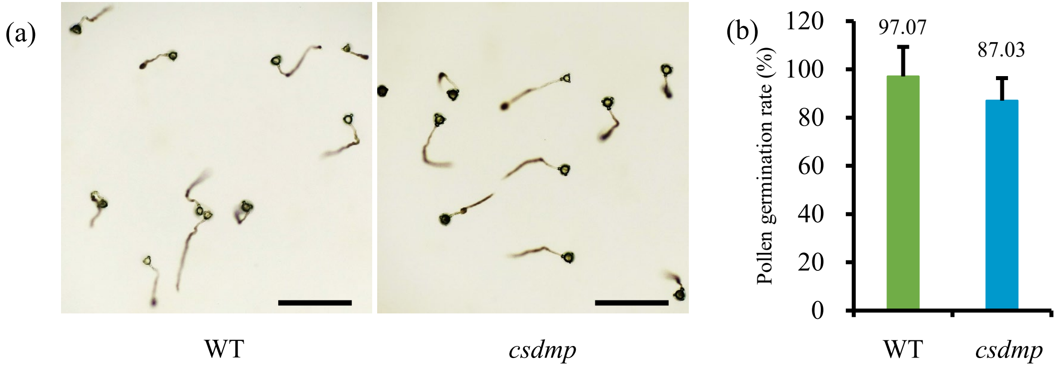

Supplemental Figure S4. The pollen germination characteristics of *csdmp* mutant compared to WT (wild type). (a) The mature pollen (at 0DBF stage) germination phenotypes between WT (left) and *csdmp* mutant (right). Scale bar = 500 µm. (b) The mature pollen (at 0DBF stage) germination rate of both WT and *csdmp* mutant. The number of pollen grains is 888 for WT and 640 for *csdmp* mutant. The values were the means ± s.d..

Supplemental Table S1. *CsDMP*-like genes in cucumber

Gene ID	Total Score	Query Cover	E value	Identity	Accession
CsDMP (CsaV3_1G028660)	271	90%	3e-93	66.67%	XP_004146729.1
CsDMP7 (CsaV3_2G034450)	136	84%	3e-40	41.81%	XP_011649980.1
CsDMP6 (CsaV3_6G050420)	144	85%	2e-43	40.57%	XP_004134946.1
CsDMP2 (CsaV3_3G013980)	106	80%	1e-28	36.14%	XP_004134465.3
CsDMP1 (CsaV3_3G013970)	97.8	83%	1e-25	34.30%	XP_004134464.1

Supplemental Table S2. The information of haploids from different crosses

Event		Type of female	Total seeds	Haploids
CCMC ×	*csdmp-1*	local cultivar	922	2
	csdmp-2		466	2
3407 ×	*csdmp-1*	inbred line	313	1
	csdmp-2		528	1
3548-1 ×	*csdmp-1*	inbred line	391	2
	csdmp-2		469	1
3569-8 ×	*csdmp-1*	inbred line	562	0
	csdmp-2		238	1
3592-5 ×	*csdmp-1*	inbred line	345	2
	csdmp-2		472	0
3610-4 ×	*csdmp-1*	inbred line	782	0
	csdmp-2		523	2
3611-2 ×	*csdmp-1*	inbred line	541	1
	csdmp-2		351	0
3679 ×	*csdmp-1*	inbred line	783	0
	csdmp-2		338	1
3681 ×	*csdmp-1*	inbred line	631	3
	csdmp-2		364	1
5569-9 ×	*csdmp-1*	inbred line	477	0
	csdmp-2		391	1
6101-4 ×	*csdmp-1*	inbred line	672	0
	csdmp-2		538	2
6101-11 ×	*csdmp-1*	inbred line	300	1
	csdmp-2		568	0

Supplemental Table S3. Primers used in this study

**Primers for CRISPR**	**Primer sequence (5'-3')**
CsDMP-BsF	ATATATGGTCTCGATTGGCTTCTGCCGTCGGTCTCCGTT
CsDMP-F0	TGGCTTCTGCCGTCGGTCTCCGTTTTAGAGCTAGAAATAGC
CsDMP-R0	AACCTGGGCGTCACGATCCCGTCAATCTCTTAGTCGACTCTAC
CsDMP-BsR	ATTATTGGTCTCGAAACCTGGGCGTCACGATCCCGTCAA
**Primers for RT-qPCR**	**Primer sequence (5'-3')**
CsDMP-qPCR-F	GTTCACGCCATTATGTCTGTAA
CsDMP-qPCR-R	GCCATACAACCAATACCATATC
CsUBI-qPCR-F	CACCAAGCCCAAGAAGATC
CsUBI-qPCR-R	TAAACCTAATCACCACCAGC
**Primers for subcellular localization**	**Primer sequence (5'-3')**
CsDMP-SL-F	CAAATCGACTCTAGAAAGCTTATGGACGAACACACAGTAACCTC
CsDMP-SL-R	CATGGTACCGGATCCACTAGTATTAGCCATACAACCAATACCATAT

Supplemental Materials and Methods

Supplemental Method S1. Plant materials and growth conditions

The *csdmp* mutants used in this study were in the "xintaimici" cucumber (*Cucumis sativus*) cultivar background. One local cultivar (CCMC) and 11 inbred lines (listed in Table S2) were used as female parent or male parent in crosses. All cucumber plants were grown in a growth chamber (28°C, 16h light /18°C, 8h dark photoperiod) or greenhouse, and the *Nicotiana benthamiana* seedlings were grown in a growth chamber (22°C, 16h light /8h dark photoperiod), in China Agricultural University, Beijing, China.

Supplemental Method S2. Vector construction and plant transformation

The CRISPR-Cas9 vector pKSE402 was provided by Prof. Sanwen Huang. The two CRISPR/Cas9 vectors targeted sites at the exon of *CsDMP* gene were designed using the online tool CRISPR-P v2.0 (http://crispr.hzau.edu.cn/CRISPR2/), and then incorporated into vector pKSE402 at *BsaI* bestriction endonuclease site by using vector pCBC-DT1T2 as template. The resulting vector was validated by sequencing and then introduced into Agrobacterium (*Agrobacterium tumefaciens*) strain GV3101. The CRISPR/Cas9 vector targeting *CsDMP* gene was transformed into "xintaimici" (Wang et al., 2014; Hu et al., 2017; Cheng et al., 2023). The primers were listed in Supplemental Table S3.

Supplemental Method S3. Pollen viability determination and germination assay

Mature fresh pollen samples of WT (wide type, "xintaimici") and *csdmp* mutants at 0DBF stage were collected at 8:00 am-9:00 am in the greenhouse, and each collection remained three biological replicates. Pollen viability was examined via pollen viability stain solution (I-KI); after 5 min, the viability was checked by microscope examination. The pollen germination assay was performed on the germination media (10% (w/v) sucrose, 0.1% (w/v) yeast extract, 0.01% (w/v) boric acid, 10 mM $CaCl_2$, 50 μM KH_2PO_4, 15% (v/v) polyethylene glycol 4000). After incubation at 28 °C for 2 h, the pollen germination rate was examined by using microscope (Li et al., 2021).

Supplemental Method S4. Haploid induction and identification

After two selfing generations of *csdmp* mutants, the T2 progenies, which are homozygous in both the mutant sites of *CsDMP* and the genome region of EGFP marker insertion, were selected as male or female parent for haploid induction. The haploids were screened from selfing or cross progenies via green fluorescence by a hand-held lamp (LUYOR-3260 flashlight) or flow cytometry. The offspring without green fluorescence were the putative haploids and then further confirmed by molecular marker and ploidy analysis.

Supplemental Method S5. Flow cytometry

Fresh young leaves (0.5 g) were chopped with a razor blade in 2 ml lysis buffer (15 mM

Tris, 2 mM disodium EDTA, 0.5 mM spermine tetrahydrochloride, 0.1% (v/v) Triton X-100 and 15 mM β-mercaptoethanol (pH 7.5)) (Zhong et al., 2020). The homogenate was centrifuged (1,000 r.p.m. at 4 °C, 5 min) and then filtered through a 40μm nylon filter to collect the nuclei. The collected nuclei were stained with propidium iodide for 20 min in dark condition, the samples ploidy was analyzed using a flow cytometer (Partec CyFlow Space). Data were acquired and analyzed with the FloMax software. The first signal peak of diploid control was set at ~100 (FL4 value). Samples with the first signal peak at ~50 (FL4 value) were regarded as haploids.

Supplemental Method S6. Phylogenetic analysis

The full-length amino acid sequences of *CsDMP*-Like genes were downloaded from Cucurbit Genomics Database (http://cucurbitgenomics.org/). The full-length amino acid sequences of *DMP* homologues of maize (*Zea mays*), tomato (*Solanum Lycopersicon*), watermelon (*Citrullus lanatus*), *Arabidopsis* (*Arabidopsis thaliana*) and *Medicago truncatula* were downloaded from NCBI (https://www.ncbi.nlm.nih.gov/). Multiple sequence alignment analysis was carried out using ClustalW, and phylogenetic tree was generated by MEGA 11 (neighbor-joining (NJ) method with default parameters: bootstrap method setting to 1000, Poisson model, and complete deletion) (Yin et al., 2022).

Supplemental Method S7. Subcellular localization

The full-length coding sequence of *CsDMP* without the stop codon was cloned into pSuper1300::EGFP vector. The primers were listed Supplemental Table S3. The pSuper::CsDMP-EGFP and the plasma membrane marker CD-1007-mCherry (Nelson et al., 2007) plasmids were subsequently transformed into Agrobacterium strain GV3101, respectively. After protein co-expression of CsDMP and CD-1007-mCherry for three days in young *Nicotiana benthamiana* leaves via Agrobacterium-mediated transformation, the localization of pSuper::CsDMP-EGFP and CD-1007-mCherry marker was checked by Nikon A1 laser confocal scanning microscope. The images were acquired with confocal microscope software NIS-Elements. Laser excitations were at 488 nm for GFP signal and at 561 nm for mCherry signal.

Supplemental Method S8. RT-qPCR Analysis

The sample RNA was extracted using the Quick RNA Isolation Kit (Huayueyang, Beijing, China) and then applied to synthesize the first-strand cDNA by the FastKing gDNA Dispelling RT SuperMix (TianGen Biotech, Beijing, China). RT-qPCR was performed using the UltraSYBR Mixture (Low ROX) (Cwbio, Beijing, China) on an Applied Biosystems 7500 real-time PCR system (Applied Biosystems, Foster City, CA, USA). The *UBIQUITIN EXTENSION PROTEIN* (*UBI*) gene was used as a reference gene. The primers were listed Supplemental Table S3. Three biological and three technical replicates were carried out for expression dynamics analysis (Yin et al., 2022).

Supplemental References

Cheng Z, Liu X, Yan S, Liu B, Zhong Y, Song W, Chen J, Wang Z, Che G, Liu L, Ying A, Lv H, Han L, Li M, Zhao J, Xu J, Yang Z, Zhou Z, Zhang X (2023) Pollen tube emergence is mediated by ovary-expressed ALCATRAZ in cucumber. Nat Commun **14:** 258

Hu B, Li D, Liu X, Qi J, Gao D, Zhao S, Huang S, Sun J, Yang L (2017) Engineering Non-transgenic Gynoecious Cucumber Using an Improved Transformation Protocol and Optimized CRISPR/Cas9 System. Molecular Plant **10:** 1575-1578

Li Y, Lin Z, Yue Y, Zhao H, Fei X, E L, Liu C, Chen S, Lai J, Song W (2021) Loss-of-function alleles of *ZmPLD3* cause haploid induction in maize. Nat Plants **7:** 1579-1588

Nelson BK, Cai X, Nebenführ A (2007) A multicolored set of *in vivo* organelle markers for co-localization studies in Arabidopsis and other plants. The Plant Journal **51:** 1126-1136

Wang H, Sui X, Guo J, Wang Z, Cheng J, Ma S, Li X, Zhang Z (2014) Antisense suppression of cucumber (*Cucumis sativus* L.) sucrose synthase 3 (*CsSUS3*) reduces hypoxic stress tolerance. Plant Cell and Environment **37:** 795-810

Yin S, Li S, Gao Y, Bartholomew ES, Wang R, Yang H, Liu C, Chen X, Wang Y, Liu X, Ren H (2022) Genome-Wide Identification of *YABBY* Gene Family in Cucurbitaceae and Expression Analysis in Cucumber (*Cucumis sativus* L.). Genes (Basel) **13:** 467

Zhong Y, Chen B, Li M, Wang D, Jiao Y, Qi X, Wang M, Liu Z, Chen C, Wang Y, Chen M, Li J, Xiao Z, Cheng D, Liu W, Boutilier K, Liu C, Chen S (2020) A *DMP*-triggered in vivo maternal haploid induction system in the dicotyledonous *Arabidopsis*. Nat Plants **6:** 466-472
